# Telescreening satisfaction: disparities between individuals with diabetic retinopathy and community health center staff

**DOI:** 10.1186/s12913-022-07500-w

**Published:** 2022-02-08

**Authors:** Xiaofeng Zhu, Yi Xu, Lina Lu, Haidong Zou

**Affiliations:** 1 Department of Preventative Ophthalmology, Shanghai Eye Disease Prevention and Treatment Center/Shanghai Eye Hospital, Shanghai, China; 2 Department of Ophthalmology, Shanghai General Hospital, Shanghai Jiao Tong University School of Medicine, Shanghai, China; 3 Shanghai Engineering Research Center of Precise Diagnosis and Treatment of Eye Diseases, Shanghai, China; 4 National Clinical Research Center for Eye Diseases, Shanghai, China; 5 Shanghai Key Laboratory of Fundus Diseases, Shanghai, China

**Keywords:** Satisfaction, Telescreening, Vision-threatening diabetic retinopathy, Community health centers

## Abstract

**Background:**

The success of telescreening and the management of diabetic retinopathy (DR) in communities depends on stakeholder satisfaction, including both individuals with diabetes and community health center (CHC) staff. In this study, we investigated the satisfaction of both individuals with vision-threatening DR (VTDR) and CHC staff within the Shanghai Eye Disease Study (SEDS) comprehensive system for managing diabetic eye diseases at the primary care level.

**Methods:**

The cross-sectional survey of patients receiving the service included 3,817 respondents with VTDR and focused on their satisfaction with the SEDS system, including the telescreening process, speed of feedback, interpretation of results, increased awareness of related diseases, and eye care services. The survey of the providers included 234 CHC staff respondents and focused on their satisfaction and the main barriers encountered during the implementation of the system. Sociodemographic characteristics and perceived barriers related to satisfaction were identified by conducting univariate and multivariate logistic regression analyses.

**Results:**

The overall satisfaction of service recipients was 96.0%, and 75.8% of them were willing to undergo future telescreening for DR. The convenience of telescreening, organization of telescreening, and improvement in related disease awareness significantly correlated with satisfaction. Only 48.3% of the providers were satisfied with the SEDS system. The most frequently mentioned barriers to the development of the system were the inadequate levels of staffing (particularly technical staff), insufficient funding, and incomplete information transmission systems.

**Conclusions:**

Disparities between high patient satisfaction and low provider satisfaction with the SEDS system were mainly related to the current weak level of ophthalmic expertise in the CHCs and the low awareness of screening for diabetic eye diseases among both patients and providers.

**Supplementary Information:**

The online version contains supplementary material available at 10.1186/s12913-022-07500-w.

## Background

The prevalence of chronic diseases, such as diabetes and hypertension, continues to rise along with increasing population longevity and lifestyle transformation [1, 2]. Individuals with chronic diseases require high-quality, accessible health care with disease management that is comprehensive, continuous, and personalized [3]. Although community-based primary care health services have existed in the People’s Republic of China (China) for more than 10 years, they have not achieved development goals and exhibit significant regional disparity [4, 5]. Moreover, the level of health care in community health centers (CHCs) is generally low owing to inadequate levels of staffing, low professional quality, and a shortage of medical resources [6, 7].

Against the background of its Healthy China strategy, the Chinese government has initiated reforms to its three-tier hierarchical medical system, which includes primary (community), general (secondary), and specialized (tertiary) hospitals. CHCs—a critical component of the primary health care system—are responsible for disease prevention and control, improvement of the general quality of life among the Chinese population, and promotion of health equality [8]. As such, CHCs undertake tasks such as the screening and diagnosis of diseases, referrals to hospitals, and the implementation of optimal strategies for the management of diseases based on intervention classification and outcomes. For example, community screening for individuals with diabetes can prevent visual impairment and blindness caused by diabetic retinopathy (DR) [9, 10]. However, although regular community DR screening is common in wealthy nations in Europe and the United States [11, 12], implementation is challenging in low- and middle-income countries owing to the increasing prevalence of diabetes, lack of eye care resources, and limited access to quality, affordable eye care [10, 13, 14]. Furthermore, most of the community management guidelines for DR are for higher-income countries with higher available resources, but no standardized guidelines exist in most developing countries with limited resources, such as China [15, 16].

In 2015, the Shanghai Diabetic Eye Study (SDES) initiated the development of a comprehensive management system for diabetic eye diseases that focuses on telescreening, referrals, and eye health management [17, 18]. It meets the eye care needs of individuals with diabetes at the community level, including the early screening, diagnosis, and intervention of DR. The success of the system depends on the satisfaction of patients receiving the services. However, the satisfaction of CHC staff—who are responsible for DR telescreening, intervention classification, and disease management—with the system is also an important factor in judging whether China’s health care system reform efforts are successful [19]. Service providers (CHC staff) and service recipients (patients) were regularly surveyed during the SDES project and seldom shared the same perspective regarding satisfaction.

In this study, we investigated the satisfaction of both service providers and recipients with the SEDS management system to understand the needs of patients with DR and clarify the difficulties faced by CHC staff. Our study has implications for scholars, public health policymakers, and stakeholders for the development of policies to promote a comprehensive management system of diabetic eye diseases at the primary care level and improve the usage of community eye care services among the targeted population.

## Methods

### Shanghai Diabetic Eye Study

The SDES was conducted from 2015 to 2017. A total of 211,469 patients with diabetes, aged 35 years and above, in 240 CHCs were enrolled in the study. All participants were telescreened for diabetic eye diseases by qualified optometrists and general practitioners in the CHCs, who had completed training in the SEDS comprehensive management system for diabetic eye diseases, including visual acuity assessment, autorefraction, and non-mydriatic fundus photography. A desktop digital 45° to 55° non-mydriatic retinal camera was used to obtain color retinal photographs of the early treatment DR study standard field 1 (centered on the optic disc) and field 2 (centered on the macula) for each eye [20]. Ophthalmologists in hospitals set up a remote retinal reading group and were responsible for the DR grading. Participants diagnosed with vision-threatening diabetic retinopathy (VTDR) were referred to general (secondary) and specialized (tertiary) hospitals for further diagnosis and treatment. Patients with diabetes were classified as “management” in the CHCs, based on patient diagnosis after telescreening and referrals [17, 18].

## Study subjects

### Recipients (individuals with DR)

Among the participants, all patients were from the SDES project. The inclusion criteria were as follows for individuals with diabetes: (1) completed DR telescreening; (2) assessed with VTDR by remote retinal image reading; (3) received classification management of diabetic eye diseases; and (4) independently completed our study’s self-report questionnaire. The eligible participants were informed about the investigation time and place, and a door-to-door survey was conducted for participants with limited physical mobility.

### Providers (CHC staff)

All 240 CHC staff were involved in establishing and implementing the SEDS comprehensive management system. They were responsible for telescreening and the classification of interventions that the CHCs, general (secondary), and specialized (tertiary) hospitals perform for diabetic eye diseases. The leader of the SDES project developed the satisfaction self-report questionnaire based on staff feedback.

This study was approved by the institutional review board of Shanghai General Hospital and adhered to the tenets of the Declaration of Helsinki. Written informed consent was obtained from all the participants and from a legal guardian or next of kin in case of illiterate participants prior to enrollment.

### Satisfaction self-report questionnaire development

Key informant interviews were conducted with both patients with DR (service recipients) and CHC staff (service providers) to assess their views and satisfaction regarding all aspects of the SEDS comprehensive management system prior to questionnaire development. The survey for individuals with DR focused on sociodemographic characteristics and perceived satisfaction with the system, including the following: the convenience of telescreening; organization of the community telescreening process; speed of feedback; interpretation of telescreening results; improvement in awareness of related diseases; eye care services in the communities. The survey of CHC staff focused on the current status of prevention and treatment of diabetic eye diseases in the communities, the main barriers currently encountered in the SEDS system, and challenges to address in the long-term development of the system. The satisfaction questionnaire includes a 5-point Likert scale ranging from 1 (*very unsatisfied*) to 5 (*very satisfied*) and multiple-choice questions.

### Data analysis

From January 1 to March 31, 2018, 3,817 patients (3817/4140, 92.2% completion rate) and 234 CHCs (234/240, 97.5% completion rate) completed a self-report questionnaire of satisfaction with the SEDS comprehensive management system. The data were analyzed using IBM® SPSS® Statistics 22, and a *p* value < 0.05 was considered statistically significant. The responses dichotomized between “satisfied” or “very satisfied” were considered an indication of satisfaction with the system, while others were considered an indication of dissatisfaction with the system.

First, descriptive statistical analysis was applied to analyze the following: the general condition of individuals with DR; the current situation of prevention and treatment of diabetic eye diseases in the CHCs; the overall satisfaction of both service recipients and providers with the SEDS comprehensive management system; their opinions and suggestions. Second, univariate associations with variables of the overall satisfaction of the system were tested using the chi-squared test or independent-samples *t*-test. Furthermore, the variables with significant univariate associations were included in a multivariate logistic regression model to calculate the odds ratios and 95% confidence intervals of the variables related to the overall satisfaction with the comprehensive management system.

## Results

### Satisfaction of service recipients

#### Sample characteristics

The patients’ ages ranged from 35 to 93 years, with an average age of 66.8 years (standard deviation: 8.2); 82.7% were older than 60 years. More than 40% of the participants had only a primary school education or were illiterate. The sociodemographic data of the patients are presented in Table 1.

**Table 1:** Descriptives and satisfaction of the people with diabetic retinopathy with the System

	**Satisfaction**					**Total(%)**	**Univariate**^**a**^	
	**Very unsatisfied**	**Unsatisfied**	**No Opinion/ Don’t Know**	**Satisfied**	**Very satisfied**			
							**χ**^**2**^**Value**	***P* Value**
**District (No. [%])**							2.66	0.26
Urban area	0 (0)	7 (0.6)	34 (3.1)	756 (68.9)	301 (27.4)	1098 (28.8)		
Suburb area	1 (0.1)	14 (0.9)	46 (2.9)	1159 (74.1)	344 (22.0)	1564 (41.0)		
Semi-urban suburb area	1 (0.1)	4 (0.3)	45 (3.9)	850 (73.6)	255 (22.1)	1155 (30.3)		
**Gender (No. [%])**							1.52	0.22
Male	0 (0)	15 (0.9)	51 (3.1)	1195 (71.6)	408 (24.4)	1669 (43.7)		
Female	2 (0.1)	10 (0.5)	74 (3.4)	1570 (73.1)	492 (22.9)	2148 (56.3)		
**Age (No. [%])**							7.80	0.10
< 50	0 (0)	2 (2.3)	1 (1.2)	73 (84.9)	10 (11.6)	86 (2.3)		
50 ~ < 60	0 (0)	1 (0.2)	18 (3.1)	420 (73.3)	134 (23.4)	573 (15.0)		
60 ~ < 70	0 (0)	13 (0.8)	51 (3.0)	1222 (71.2)	430 (25.1)	1716 (45.0)		
70 ~ < 80	2 (0.2)	9 (0.7)	45 (3.7)	882 (72.8)	274 (22.6)	1212 (31.8)		
≥ 80	0 (0)	0 (0)	10 (4.3)	168 (73.0)	52 (22.6)	230 (6.0)		
**Marital Status (No. [%])**^**b**^							2.92	0.09
With a partner	1 (0)	21 (0.6)	104 (3.0)	2510 (72.5)	824 (23.8)	3460 (90.6)		
Without a partner	1 (0.3)	4 (1.1)	21 (5.9)	255 (71.4)	76 (21.3)	357 (9.4)		
**Education level (No. [%])**							7.39	0.60
Illiteracy	0 (0)	6 (1.7)	15 (4.2)	262 (72.8)	77 (21.4)	360 (9.5)		
Primary school	1 (0.1)	9 (0.7)	41 (3.3)	893 (72.4)	289 (23.4)	1233 (32.4)		
High school	1 (0.0)	8 (0.4)	62 (3.1)	1466 (72.3)	491 (24.2)	2028 (53.3)		
College degree or above	0 (0)	2 (1.1)	7 (3.8)	137 (73.7)	40 (21.5)	186 (4.9)		

^a^Chi-squared test or independent-samples t test for univariate associations with variables of the overall satisfaction of the System

^b^With a partner included married and domestic partnership, and without a partner included single, separated, divorced and widowed

#### Satisfaction analysis

The overall satisfaction with the SEDS system reported by patients receiving the service (recipients) was 96.0% (3665/3817); 75.8% (2892/3817) of them were willing to continue telescreening for diabetic eye diseases in the communities the following year. No significant difference was observed in the overall satisfaction with the system among individuals with DR living in different areas or with different age, sex, marital status, and educational levels (Table 1).

According to the satisfaction survey regarding all aspects of the SEDS system (Table 2), the following were significantly associated with overall satisfaction (*p* < 0.05): the convenience of community telescreening (95.8%); organized process of telescreening (92.0%); environment (94.2%); staff (95.6%); feedback speed (89.1%) and interpretation (92.0%) of telescreening results; improvement in the awareness of the related diseases (96.0%); satisfaction with eye care services provided by the CHCs (96.1%). In the multivariate logistic regression model, the convenience of community telescreening, organization of telescreening processes, and improvement in the related disease awareness significantly correlated with overall satisfaction (Table 2).

**Table 2:** Description of satisfaction with various phases of the telescreening in people with diabetic retinopathy

		**Satisfaction(No. [%])**					**Univariate Analysis**^**a**^		**Multivariate Analysis**^**b**^	
		**Very unsatisfied**	**Unsatisfied**	**No Opinion / Don’t Know**	**Satisfied**	**Very satisfied**	**χ**^**2**^** Value**	***P* Value**	**OR (95% CI)**	***P* value**
**Convenience of telescreening in the community**		3 (0.1)	25 (0.7)	131 (3.4)	2722 (71.3)	936 (24.5)	121.9	< 0.001	3.59 (2.09–6.15)	< 0.001
**Telescreening field**	**The process was reasonable and orderly**	7 (0.2)	41 (1.1)	255 (6.7)	2814 (73.7)	698 (18.3)	100.9	< 0.001	2.91 (1.69–5.00)	< 0.001
	**The layout was reasonable, and the environment was neat**	1 (0.0)	30 (0.8)	188 (4.9)	2860 (74.9)	736 (19.3)	36.7	< 0.001	-	-
	**The staff’s instruction was clear, and attitude was kind**	1 (0.0)	25 (0.7)	138 (3.6)	2831 (74.2)	818 (21.4)	41.4	< 0.001	-	-
**Speed of feedback**		16 (0.4)	164 (4.3)	236 (6.2)	2729 (71.5)	671 (17.6)	32.7	< 0.001	-	-
**Interpretation of telescreening results**		4 (0.1)	55 (1.4)	245 (6.4)	2751 (72.1)	761 (19.9)	74.7	< 0.001	-	-
**Improving awareness of related diseases**		2 (0.1)	25 (0.7)	125 (3.3)	2762 (72.4)	899 (23.6)	106.6	< 0.001	2.38(1.35–4.18)	0.003
**Eye care service in the community**		2 (0.1)	25 (0.7)	124 (3.3)	2762 (72.5)	899 (23.6)	101.6	< 0.001	-	-

*OR* Odds ratio

^a^Chi-squared test for univariate associations with variables of the overall satisfaction of the System

^b^Variables with significant univariate associations included in a multivariate logistic regression model

#### Satisfaction of providers

##### Sample characteristics

Among the 234 CHCs, only 49 (20.9%) had independent ophthalmic clinics; 78 (33.3%) had ophthalmic and otolaryngologic comprehensive clinics. Further, 47 (20.1%) had ophthalmologists from hospitals regularly providing eye care services, and 89 (38.0%) had no eye care service.

The number of CHCs without eye care services in the suburban area was much higher than that in the urban and semi-urban areas (Supplemental Table 1). The prevention and treatment of diabetic eye diseases were integrated into the medical services of the family doctor teams in the 66 CHCs (28.2%) and general practice services in 80 CHCs (34.2%). Moreover, 160 CHCs (68.4%) signed bilateral cooperation agreements with general (secondary) and specialized (tertiary) hospitals (Supplemental Table 2).

#### Satisfaction analysis

Among the CHCs, 113 (48.3%) were satisfied with the SEDS comprehensive management system (Table 3). Significant differences were found in satisfaction among the CHCs in different areas (χ^2^ = 45.0, *P* < 0.001); the satisfaction in the semi-urban areas was the highest (78.4%), and that in the suburban areas was the lowest (25.6%). Only 99 CHCs (42.3%) considered that it was necessary to conduct diabetic eye disease telescreening at the community level for individuals with diabetes, which significantly correlated with overall satisfaction with the system (*P* < 0.001). Furthermore, telescreening and management of diabetic eye diseases in the community were considered to incorporate routine work in 119 CHCs (51.3%) and add to the performance review of 128 CHCs (54.7%) (Table 4).

**Table 3:** Description of satisfaction with the System among the community health service centers’ staff

District	Satisfaction (No. [%])				
	**Very unsatisfied**	**Unsatisfied**	**No Opinion/ Don’t Know**	**Satisfied**	**Very satisfied**
**Urban area**	4 (5.4)	31 (41.9)	6 (8.1)	30 (40.5)	3 (4.1)
**Suburb area**	11 (12.8)	49 (57.0)	4 (4.7)	17 (19.8)	5 (5.8)
**Semi-urban suburb area**	0 (0)	6 (8.1)	10 (13.5)	52 (70.3)	6 (8.1)
**Total**	15 (6.4)	86 (36.8)	20 (8.5)	99 (42.3)	14 (6.0)

**Table 4:** Necessity of screening for diabetic eye diseases in the communities and correlation with the satisfaction

	**Telecreening for diabetic eye disease**			**Correlation with the overall satisfaction**	
	**Necessary**	**Don’t Know**	**Unnecessary**	**χ**^**2**^** Value**	***P* Value**
**Telecreening in the community**	99(42.3)	23(9.8)	112(47.9)	117.60	< 0.001
**Incorporate routine work**	119(51.3)	20(8.6)	93(40.1)	1.09	0.58
**Added to performance review**	128(54.7)	28(12.0)	78(33.3)	0.75	0.69

### CHC recommendations

A total of 161 CHCs (68.5%) suggested that diabetic eye disease telescreening and management could be merged with other community health work. Among these, 79 (49.1%) believed that it could be merged with the ongoing management of patients with diabetes. Further, 52 (32.3%) thought that it could be merged with physical examinations for older adults, and the remaining 30 (18.6%) presumed that it could help in telescreening for diabetic eye diseases in the outpatient clinic on weekdays (Supplemental Fig. 1).

**Fig. 1:**
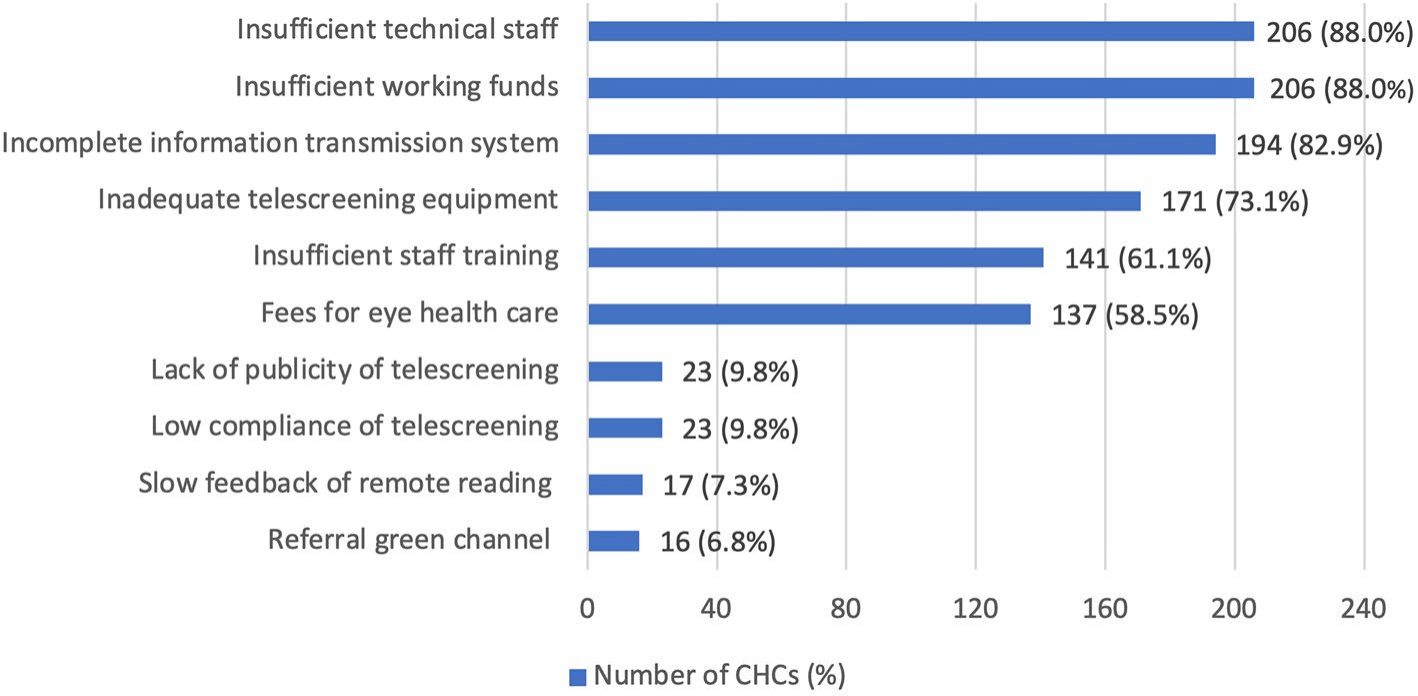
Potential difficulties in the long-term development of telescreening for diabetic eye disease (*N* = 234)

Regarding the difficulties in the long-term development of the SDES comprehensive management system (Fig. 1), the most frequently mentioned barriers were the following: inadequate number of staff (particularly, technical staff) for telescreening in the communities; insufficient funding; an incomplete information transmission system for telescreening. Inadequate telescreening equipment, insufficient staff training, and fees for eye health care were also frequently mentioned barriers. Other barriers included the following: the lack of publicity and promotion of telescreening for diabetic eye diseases in the communities; the low compliance of eye health screening among individuals with diabetes; the slow feedback of remote reading; the lack of a dedicated green channel for referral patients to reduce time and costs and facilitate the use of the referral system.

## Discussion

As telescreening for DR has matured, CHCs have become more capable of providing screening, diagnosis, intervention classification, management, and follow-up for diabetic eye diseases. This has significantly improved the coverage rate of individuals with diabetes and the accessibility of eye care services [21]. Although telescreening for DR in communities has achieved significant advantages owing to the comprehensive management system in Shanghai, many challenges still exist.

The first challenge is to determine whether stakeholders—specifically, individuals with diabetes and CHC staff—were satisfied with the comprehensive management system. A satisfaction survey is a quantitative tool for obtaining information directly from service recipients and providers [19]. Higher recipient satisfaction can improve their willingness to participate in future telescreening for diabetic eye diseases while increasing the popularity of the system. Higher provider satisfaction is closely related to implementation efficiency and the long-term development of a comprehensive management system [22].

In this study, we investigated the satisfaction of both service recipients and providers participating in the SEDS comprehensive management system of diabetic eye diseases in Shanghai. The findings are as follows.

First, most individuals with DR were satisfied with telescreening for diabetic eye diseases in their community. Additionally, they were willing to participate in the telescreening the following year, which was consistent with other studies that reported higher satisfaction in individuals with diabetes for DR telescreening [19, 23]. Overall satisfaction was significantly related to the actual experience of individuals with DR during the telescreening process. The convenience of telescreening, the organization of the telescreening process, and the improvement in awareness of related diseases were significantly associated with the overall satisfaction with the SEDS comprehensive management system among individuals with DR. The fact that telescreening for DR was free also contributed to the high satisfaction of service recipients. Similar to findings in other countries, this study’s results demonstrated that individuals with diabetes believed that DR telescreening, compared with the traditional fundus examination, was more convenient and faster. It reduced the time and cost of visiting doctors and increased their understanding of their disease. Further, it provided support for further clinical diagnosis and treatment [23–25]. Additionally, the common reasons for dissatisfaction in all developing countries are inconvenient communication facilities and interrupted network support. The transfer of information in real time is often impossible. Telescreening data are stored and transferred at a convenient time to hospitals for diagnosis; hence, the patients are not promptly informed of their examination results and interventions. Therefore, the following are crucial to improve patients’ satisfaction with the SEDS system: further optimization of the DR telescreening process; a comfortable telescreening environment; timely feedback of telescreening results and referral recommendations; strengthening the training of community staff and education about related diseases for individuals with DR.

Second, the overall satisfaction of the CHCs with the comprehensive management system was not ideal. Less than half of the CHCs were satisfied with the system, and satisfaction of the suburban CHCs was relatively low. According to our study, nearly two-fifths of the CHCs did not provide eye care services, and this situation was especially prevalent in the suburban CHCs. Thus, weaker ophthalmic clinical competence in the suburban CHCs caused a heavier telescreening workload, resulting in lower satisfaction with the system. Through the establishment of the SEDS comprehensive management system, one-third of the CHCs included the prevention and treatment of diabetic eye diseases as part of the services of general practitioners or family doctors teams, among which the CHCs in urban areas had the highest proportion. In China, family doctors are mainly registered general practitioners, and some qualified village doctors are also considered family doctors. Family doctor teams—including family doctors, community nurses, public health physicians, and village doctors—provided health management services to community residents [26]. A well-implemented family doctor system has been positively related to better health outcomes and health care cost containment in practice [27]. Therefore, including telescreening for DR into the routine work of family doctors teams should significantly help promote the system and improve the prevention and treatment effect. Nearly 70% of CHCs signed cooperation agreements with general (secondary) and specialized (tertiary) hospitals, with the highest proportion in urban areas. However, nearly half of the CHCs complained that telescreening for diabetic eye diseases at the community level was not necessary at present, considering the inadequate number of technical staff, inadequate funding, and the lack of awareness of the DR disease. Consequently, staff enthusiasm for their work was negatively affected, and this was significantly associated with overall satisfaction with the system. Meanwhile, many of the CHCs suggested merging diabetic eye disease telescreening with the disease management of individuals with diabetes, physical examinations for older adults, or routine outpatient clinics on weekdays.

Furthermore, several problems must be solved for the long-term successful development of the SEDS comprehensive management system. First, the inadequate number of CHC staff—especially those who can operate the telescreening equipment, particularly non-mydriatic fundus photography—must be addressed. Hence, training for CHC staff should be increased. Second, insufficient funding affects project implementation and staff motivation. Third, inadequate telescreening equipment affects screening efficiency; the improvement of the information management system of telescreening, increased publicity and promotion of the system, and improvement of disease awareness of community residents must be addressed. Hospitals should improve the speed of remote reading feedback and set up a referral clinic for patients requiring further diagnosis and treatment for DR to improve compliance and satisfaction with referrals. Furthermore, personalized risk-based screening schedules should be examined to optimize workload and time in telescreening programs for diabetic eye diseases [28, 29].

This study is the first to investigate the satisfaction of service recipients and providers with telescreening for DR at the primary (community) care level. The participants, including patients with VTDR and CHC staff, were recruited from all the communities in Shanghai, and this reflects the diversity of the study participants.

Nevertheless, this study has several limitations. All participants who received the service were diagnosed with VTDR. Thus, the study lacked a control group (with mild DR or no apparent retinopathy), which may limit the applicability of the findings. Moreover, satisfaction with the system is based on participants’ recollections, but it could lead to inaccurate results owing to imperfect memory.

## Conclusions

This study found disparities between the high satisfaction of service recipients and the low satisfaction of providers with the comprehensive management system. This was mainly related to the current weak ophthalmic diagnosis and treatment level in the CHCs and the low awareness of screening for diabetic eye diseases among individuals with diabetes and the CHC staff. Therefore, it is important to improve the satisfaction of CHC staff with the system. For example, measures should be taken to establish a new performance evaluation system, strengthen staff training, improve the allocation of community ophthalmic equipment, and increase publicity, education, and financial support. The satisfaction of the SEDS comprehensive management system stakeholders—specifically, individuals with DR (recipients) and CHC staff (providers)— should be considered the critical assessment criteria to measure the effectiveness of public health projects. The purpose is to effectively improve the satisfaction of stakeholders in the reform of the community medical and health care system in China.

## Supplementary Information

**Additional file 1. **

**Additional file 2. **

**Additional file 3. **

## Data Availability

The datasets used and analyzed during this study are available from the corresponding author upon reasonable request.

## Abbreviations

CHC: Community health center
DR: Diabetic retinopathy
The system: A comprehensive management system for diabetic eye diseases
SDES: Shanghai Diabetic Eye Study
VTDR: Vision-threatening diabetic retinopathy
