# Supplementary material for: Telescreening satisfaction: disparities between individuals with diabetic retinopathy and community health center staff
Source: BMC Health Serv Res. 2022 Feb 8;22:160. doi: 10.1186/s12913-022-07500-w (PMC8822836; doi:10.1186/s12913-022-07500-w)
Supplement: Supplementary file 1 — Additional file 1. [file 12913_2022_7500_MOESM1_ESM.docx]

**Supplemental Table 1.** Different forms of ophthalmic medical service in the community health service centers

|  | **District (No. [%])** | | | **Total** |
| --- | --- | --- | --- | --- |
|  | **Urban area** | **Suburb area** | **Semi-urban suburb area** |  |
| **CHSCs** | 74 (31.6) | 86 (36.7) | 74 (31.6) | 234 |
| **Independent ophthalmic clinics** | 18 (24.3) | 7 (8.1) | 24 (32.4) | 49 (20.9) |
| **Ophthalmic and ENT comprehensive clinics** | 43 (58.1) | 20 (23.3) | 15 (20.3) | 78 (33.3) |
| **Ophthalmologists from the superior hospitals** | 19 (25.7) | 13 (15.1) | 15 (20.3) | 47 (20.1) |
| **No eye care service** | 9 (12.2) | 52 (60.5) | 28 (37.8) | 89 (38.0) |

CHSCs, community health service centers; ENT, ear, nose, and throat.
