# Supplementary figures and images for: Telescreening satisfaction: disparities between individuals with diabetic retinopathy and community health center staff
Source: BMC Health Serv Res. 2022 Feb 8;22:160. doi: 10.1186/s12913-022-07500-w (PMC8822836; doi:10.1186/s12913-022-07500-w)

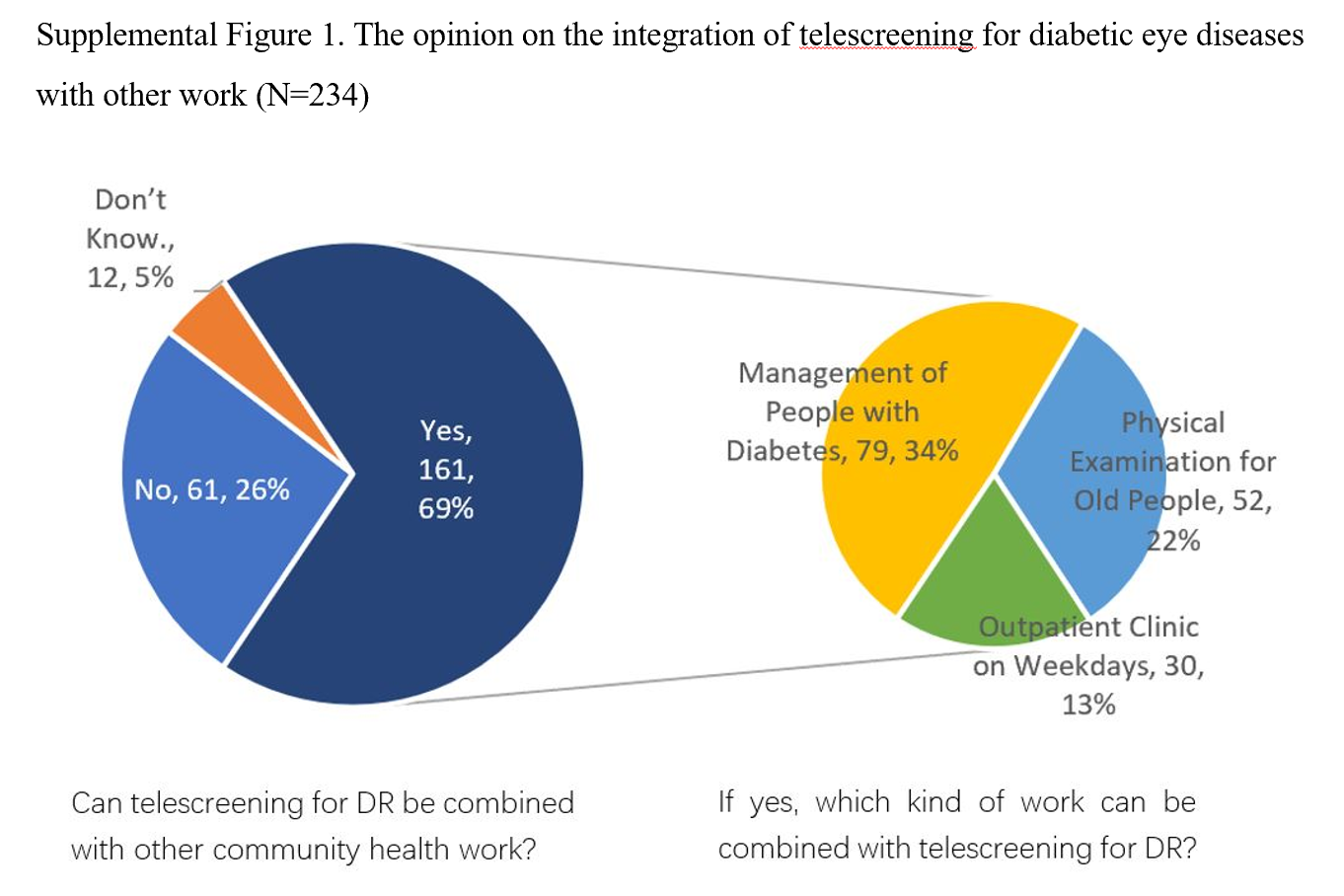

Supplement: Supplementary file 2 — Additional file 2. [file 12913_2022_7500_MOESM2_ESM.png]
