# Supplementary material for: Telescreening satisfaction: disparities between individuals with diabetic retinopathy and community health center staff
Source: BMC Health Serv Res. 2022 Feb 8;22:160. doi: 10.1186/s12913-022-07500-w (PMC8822836; doi:10.1186/s12913-022-07500-w)
Supplement: Supplementary file 3 — Additional file 3. [file 12913_2022_7500_MOESM3_ESM.docx]

**Supplemental Table 2.** Prevention and treatment of diabetic eye disease in the community health service centers

| **Prevention and treatment of diabetic eye disease in community** | **District (No. [%])** | | | **Total** |
| --- | --- | --- | --- | --- |
|  | **Urban area** | **Suburb area** | **Semi-urban suburb area** |  |
| **CHSCs** | 74 | 86 | 74 | 234 |
| **Integrated into services of general practitioner** | 41 (55.4) | 19 (22.1) | 20 (27.0) | 80 (34.2) |
| **Integrated into services of family doctor** | 32 (43.2) | 14 (16.3) | 20 (27.0) | 66 (28.2) |
| **Bilateral cooperation with the superior medical institution** | 64 (86.5) | 55 (64.0) | 41 (55.4) | 160 (68.4) |

CHSCs, community health service centers.
